# Simple quality assurance method of dynamic tumor tracking with the gimbaled linac system using a light field

**DOI:** 10.1120/jacmp.v17i5.6376

**Published:** 2016-09-08

**Authors:** Hideharu Miura, Shuichi Ozawa, Masahiro Hayata, Shintaro Tsuda, Kiyoshi Yamada, Yasushi Nagata

**Affiliations:** ^1^ Hiroshima High‐Precision Radiotherapy Cancer Center Hiroshima Japan; ^2^ Department of Radiation Oncology Institute of Biomedical and Health Science, Hiroshima University Hiroshima Japan

**Keywords:** light field, dynamic tumor tracking, quality assurance, 4D modeling

## Abstract

We proposed a simple visual method for evaluating the dynamic tumor tracking (DTT) accuracy of a gimbal mechanism using a light field. A single photon beam was set with a field size of $30\times 30\;{\text{mm}}^{2}$ at a gantry angle of 90°. The center of a cube phantom was set up at the isocenter of a motion table, and 4D modeling was performed based on the tumor and infrared (IR) marker motion. After 4D modeling, the cube phantom was replaced with a sheet of paper, which was placed perpendicularly, and a light field was projected on the sheet of paper. The light field was recorded using a web camera in a treatment room that was as dark as possible. Calculated images from each image obtained using the camera were summed to compose a total summation image. Sinusoidal motion sequences were produced by moving the phantom with a fixed amplitude of 20 mm and different breathing periods of 2, 4, 6, and 8 s. The light field was projected on the sheet of paper under three conditions: with the moving phantom and DTT based on the motion of the phantom, with the moving phantom and non‐DTT, and with a stationary phantom for comparison. The values of tracking errors using the light field were 1.12±0.72, 0.31±0.19, 0.27±0.12, and 0.15±0.09 mm for breathing periods of 2, 4, 6, and 8 s, respectively. The tracking accuracy showed dependence on the breathing period. We proposed a simple quality assurance (QA) process for the tracking accuracy of a gimbal mechanism system using a light field and web camera. Our method can assess the tracking accuracy using a light field without irradiation and clearly visualize distributions like film dosimetry.

PACS number(s): 87.56 Fc, 87.55.Qr

## I. INTRODUCTION

Breathing‐induced organ motion is one of the issues causing uncertainties during beam delivery. The greatest movement is produced in the caudal–cranial direction close to the diaphragm, such as tumors in the lower lung lobes, and upper abdominal tumors, such as liver or pancreatic tumors.^(1)^ Several methods were proposed to compensate for breathing‐induced organ motion. Breathing‐induced organ motion‐compensated treatment techniques include delivery techniques such as motion‐encompassing methods, breath holding,^(2)^ forced shallow breathing,^(3)^ respiratory gating,^(4)^ and dynamic tumor tracking (DTT).^5^, ^6^, ^7^, ^8^, ^9^, ^10^ DTT techniques were realized through reasonably accurate real‐time acquisition of the target motion of a patient using external surrogates (indirect DTT) or an internally implanted marker (direct DTT). An overview of the management of respiratory motion in radiotherapy was summarized in the report of the American Association of Physicists in Medicine (AAPM) Task Group (TG) 76.^(1)^

Indirect DTT through the Vero4DRT system has become commercially available. The DTT techniques of the Vero4DRT system require synchronization of a gimbal swing with the respiratory cycle of a patient, which is based on 4D modeling. The accuracy of indirect DTT should be verified by the model predicting the internal target position based on surrogate measurements before clinical use. Several investigators reported a high tracking accuracy of DTT through the Vero4DRT system using chamber and film measurements, even for rapidly moving patterns.^6^, ^7^, ^8^ Indirect DTT using the Vero4DRT system using the gimbal reduced the blurring effect of respiratory motion with high accuracy. The accuracy of the model predicting the internal target position based on surrogate measurement, verification of the gimbal mechanism, and patient dose verification QA, is important. As in traditional radiation therapy clinical practice, a treatment unit light field and skin marks on a patient were aligned to verify the patient position with respect to a target. Thus, the light field corresponded to the radiation field. AAPM‐TG report 40/142 recommends testing the radiation and light field agreement on a monthly basis with a tolerance of 2 mm or 1% on any side.^11^, ^12^ Here, we propose a simple visual method for evaluating the DTT accuracy of the gimbal mechanism using a light field.

## II. MATERIALS AND METHODS

### A. Vero4DRT system

Vero4DRT system (MHI‐TM2000; Mitsubishi Heavy Industries, Ltd., Tokyo, Japan, and BrainLAB, Feldkirchen, Germany) is described elsewhere.^6^, ^7^, ^8^ Vero4DRT system is equipped with a gimbaled X‐ray head for DTT, system‐specific fixed jaw, multileaf collimator (MLC), infrared (IR) camera, and image‐guided radiotherapy (IGRT) system. The MLC consists of 30 opposing pairs of 11‐cm‐thick tungsten‐alloy leaves projecting 5 mm from the isocenter and providing a maximum field size of $150\times 150\;{\text{mm}}^{2}$. The maximum leaf speed is 5 cm/s. Vero4DRT system has a fixed primary collimator positioned upstream of the MLC without movable jaws. The gantry can be rotated $\pm 185^{\circ }$ along an O‐shaped guideline at a nominal maximum speed of 7°/s, and the O‐ring can be rotated $\pm 60^{\circ }$ around its vertical axis at a nominal maximum speed of 3°/s. The IR camera can monitor IR markers on the abdominal of a patient with high accuracy, and the IR marker position, as well as a kilovoltage (KV) image, is used to perform 4D modeling. Vero4DRT system is equipped with a dual orthogonal kV imaging system mounted on the ring $\pm 45^{\circ }$ on each side of the megavolt (MV) source, and kV cone‐beam computed tomography (CBCT) using each source–detector pair. The gimbaled X‐ray head can swing along two orthogonal axes up to ±2.5°. It swings a beam up to ±41.9mm in each direction with a maximum speed of 152 mm/s from the isocenter of the isocenter plane, allowing pan and tilt motion of the linac. ExacTrac system version 3.5.3 (Brainlab AG, Feldkirchen, Germany) automated IR camera is mounted on the ceiling of a treatment room, and two orthogonal kV X‐ray imaging systems are attached to the O‐ring at 45° from the MV beam axis.

### B. Phantom study

IPlan RT Dose treatment planning system version 4.5.3 (Brainlab AG) was used for planning the design. A single photon beam was set with a field size of $30\times 30\;{\text{mm}}^{2}$ at a gantry angle of 90°.

**Figure 1:**
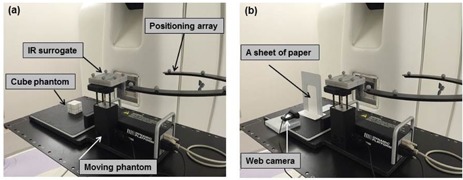
Experimental setup (a) for a single field. A cube phantom was placed on a motion table. After 4D modeling (b), a sheet of paper was placed perpendicularly on the motion table in substitution for the phantom. The web camera was placed 10 cm from the isocenter.

Figure 1 shows the experimental setup. A programmable respiratory motion table (CIRS. Inc., Norfolk, VA) was used to simulate breathing‐induced organ motion. A dynamic phantom could move based on an arbitrary input function. A motion table had one table that moved in the horizontal direction in synchrony with the table that moved in the vertical direction. Two iron markers with diameters of 2.0 mm were attached to the cube phantom substituting for gold markers. Minimum two markers are required for dynamic tracking using Vero4DRT system.

The center of the cube phantom was set up at the isocenter of the motion table that moved in the horizontal direction. An IR marker phantom was placed on the table that moved in the vertical direction motion as a surrogate signal. The motion of the surrogate was measured using the IR markers attached to the phantom monitored using the IR camera placed on the ceiling of the treatment room every 16.7 ms. A pair of orthogonal kV X‐ray cameras was rotated at gantry angles of 45° and 315° to detect the iron markers every 320 or 640 ms. The sampling interval of kV X‐ray images automatically changed to 640 ms when the velocity of the IR marker motion decreased. The acquisition times ranged from 20 to 40 s. After monitoring, the detected and predicted target positions were determined. The center of the two markers during motion was defined as the detected target position. The 4D modeling function was automatically established using ExacTrac system (Brainlab AG) after correlating the 4D motion data of the target and of the IR marker. The predicted target position was calculated from the 4D modeling function. The data on the pair of IR markers and on the iron marker was simultaneously obtained to establish the 4D modeling function. The 4D modeling function was a quadratic function of the IR marker position and velocity,^9^, ^10^

(1) $$P_{\text{predict}}=aP_{IR}^{2}+bP_{IR}+c+dv_{IR}^{2}+ev_{IR},$$

where $P_{\text{predict}}$ is the predicted target position, $P_{IR}$ is the IR marker positions, and $v_{IR}$ is the vertical velocity of the IR markers. Parameters a, b, c, d, and e were optimized by minimizing the residual errors between $P_{\text{predict}}$ and the predicted target position for each IR marker. The peak‐to‐peak amplitude of the detected target motion, as well as the mean (μ) and standard deviation (SD) of the absolute difference between the detected and predicted target positions, were automatically calculated and displayed on the screen of Vero4DRT system in the 4D modeling phase. After the 4D modeling process, DTT could be performed towards the predicted target positions from the displacements of the IR markers using the 4D modeling data. We did not use irradiation in this study.

### C. Data analysis

Our proposed method consists of the projected light field on a sheet of paper and the web camera. After 4D modeling, the cube phantom was replaced with a sheet of paper, which was placed perpendicularly, and the light field was projected on the sheet of paper. The web camera was placed 10 cm from the isocenter. The light field projected on the sheet of paper was recorded in the treatment room that was as dark as possible. The movie frames were obtained at an image size of 640×480 pixels and a frame rate of 30 fps (frames per second). The maximum dose rate in Vero4DRT system is 500 MU/min. If the monitor unit setting is assumed 100, the delivery time is 12 s. Thus, we recorded the movie during 12 s. We developed in‐house software using Visual C# (Microsoft Corporation, Redmond, WA). The obtained images were binarized to remove the noise.

**Figure 2:**
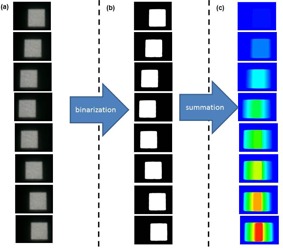
Overview of image processing: (a) the camera images are projected on a sheet of paper during IR tracking; (b) the binarized images; (c) the summation of each image.

The binarized images from each frame image were summed to compose a total summation image. Figure 2 shows the process of the summation using a light field. The square edge of the crossline (moving direction) was automatically measured. The value of the square edge on the initial frame was used as a reference value. The known distance on the paper was used to obtain the spatial resolution. A set of 1D sinusoidal motion sequences was produced by the phantom with a fixed amplitude of 20 mm and different breathing periods of 2, 4, 6, and 8 s. The IR marker motion was fixed at a peak‐to‐peak amplitude of 20 mm, and the breathing period was synchronized with the target motion. The light field was projected on the sheet of paper under three conditions: with the moving phantom and DTT based on the motion of the phantom, with the moving phantom and non‐DTT, and with a stationary phantom for comparison. We analyzed two metrics.

1. The absolute values of the differences between the reference and each frame values were considered an error of dynamic tumor tracking.
2. The absolute value of the difference between the detected and predicted target positions was considered an error of 4D modeling.

## III. RESULTS

**Figure 3:**
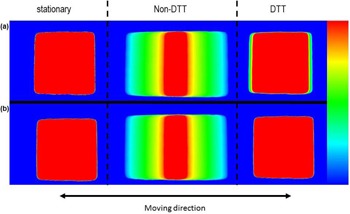
Single field for the 1D sinusoidal pattern with breathing periods of (a) 2 and (b) 8 s are under stationary, non‐DTT, and DTT conditions. DTT reduced blurring. The pixel intensities are reported in the arbitrary units.

**Figure 4:**
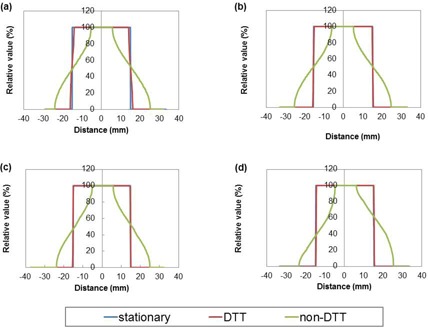
Profiles for stationary (blue line), DTT (red line), and non‐DTT (green line) conditions under different sinusoidal patterns with breathing periods of (a) 2, (b) 4, (c) 6, and (d) 8 s.

Figure 3 shows the light field measurement results for the stationary phantom, non‐DTT, and DTT at breathing period 2 and 8 sec. The stationary phantom and DTT with breathing period of 8 s had almost the same distribution, while non‐DTT increased the blurring effects of the dose distribution. DTT with a breathing period of 2 s had slight dose blurring, while non‐DTT increased the blurring effects of the distribution. Figure 4 shows the profiles for the stationary phantom, DTT, and non‐DTT with breathing periods of 2, 4, 6, and 8 s, respectively. DTT reduced the blurring effects and produced the profile curve similar to that of the stationary phantom. The effect of DTT is demonstrated visually and numerically.

The values of tracking errors using the light field were 1.12±0.72(0.00–2.23), 0.31±0.19(0.00–0.71), 0.27±0.12(0.00–0.55), and 0.15±0.09(0–0.31) mm for breathing periods of 2,

4, 6, and 8 s, respectively. The results of the 4D modeling error and light field measurements through DTT of sinusoidal patterns are summarized in Table 1. A good correlation was found between the 4D modeling error and the light field measurement results ($R^{2}=0.998$). As for the severe motion pattern (the breathing period: 2 s), the gimbaled X‐ray head cannot track the target in real time with high accuracy. The 4D modeling and tracking errors exhibit dependences on the breathing period.

**Table 1:** 95th percentile of the 4D modeling error ($4D-E_{95\%}$) and the light field measurement result.

*Breathing Period (s)*	$4D-E_{95\%}$ *(mm)*	$\text{Light}-E_{95\%}$ *(mm)*
2	1.42	2.13
4	0.37	0.61
6	0.17	0.44
8	0.13	0.31

$4D-E_{95\%}=4D$ modeling error between the detected and predicted target positions (μ+2SD); $\text{Light}-E_{95\%}=\text{tracking}$ error between the stationary and moving positions (μ+2SD).

## IV. DISCUSSION

Various DTT methods, such as CyberKnife robotic radiosurgery system with an integrated synchrony respiratory tracking system (Accuray, Sunnyvale, CA) and an real time tumor‐tracking radiotherapy RTRT system (developed by Hokkaido University School of Medicine and Mitsubishi Electronics Company Ltd., Japan) which were designed to track tumors by imaging fiducial markers implanted in the tumors, were proposed to reduce the impact of respiratory motion during beam delivery.^4^, ^13^ Several researchers performed the QA of the DTT method using a film and a phantom. In our previous study, the distance of a 90% dose profile between the stationary and DTT conditions ranged from 0.15 to 0.40 mm.^(8)^ The goal of this work is to propose a simple method that evaluates the DTT accuracy of the gimbal mechanism using a light field. The 95 percentile of the positional tracking error values analyzed using our proposed method ranged from 0.31 to 2.13 mm. Several authors reported the accuracy of DTT through Vero4DRT system using electronic portal imaging device (EPID) or video images. Akimoto et al.^(14)^ reported that the mean values of the maximum deviation of the tracking accuracy using a 2D moving phantom producing sinusoidal motion (the amplitude: ±10 mm, the breathing period: 6 s) and EPID image were 0.38, 0.49, and 0.53 mm in the pan, tilt, and 2D directions, respectively. Depuydt et al.^(15)^ reported an average 90th percentile of 0.54 mm and tracking error standard deviations of 0.20 mm for pan and 0.22 mm for tilt using a 2D moving phantom producing several sinusoidal motions (amplitude: ±10 mm, breathing period: from 2 to 12 s) and a light field. As for clinical data, Ebe et al.^(16)^ reported that the 95 percentile of the positional tracking error ranged from 0.54 to 1.55 mm using a 1D moving phantom and 16 trajectories in six patients. The images of a metal ball bearing (BB) obtained using EPID or video image are conceptually similar to the Winston–Lutz test.^(17)^ The tracking accuracy in our study is similar to that in the studies mentioned above.^13^, ^14^, ^15^, ^16^

The EPID system with Vero4DRT system has a pixel size of $0.18\times 0.18\;{\text{mm}}^{2}$ at the isocenter level and a matrix size of 1024×1024 pixels. The spatial resolution of the web camera with an image size of 640×480 pixels on the sheet of paper at the isocenter was approximately 0.1 mm in our study. This spatial resolution can gather enough data for evaluating the tracking error of DTT, because the camera has a good spatial resolution compared with EPID. AAPM TG 142 recommends that for respiratory gating, monthly QA tests should be performed owing to their functionality. However, these are not quantitative tests and thus cannot detect gradual change of the machine performance including physical accidents, component failure, and quality degradation.^(12)^ Therefore, we propose this method as simple quantitative tracking accuracy verification. As can be seen, our proposed method provides not only composite results like film dosimetry, but also the results on the measurement process for each frame. The evaluation of the gimbal mechanism using a light field suggests an advantage of low operating costs compared to film dosimetry. The web camera utilized in this study costs approximately $20.

For non‐DTT, motion caused blurring of the profile that caused an increased penumbra. In contrast, DTT dramatically reduced the blurring profile and produced a penumbra that was similar to that of the stationary phantom. The tracking error of the severe pattern (breathing period: 2 s) was increased with the breathing period and led to blurring. Slight blurring occurred for several reasons, such as delays in image processing and communication, prediction error, and mechanical response time lag.^(5)^

The limitation of this study is to be performed with a gantry angle of 90°. The weight of the gimbaled head is approximately 600 kg.^(5)^ Further investigations need to be conducted to evaluate the effect of gravity on the gimbal dynamic behavior during real‐time tracking. Mukumoto et al.^(6)^ reported that the tracking accuracy was not degraded by gantry rotation, even at an angle of 0° or 90°. This study was designed to evaluate square fields only. Further complex MLC shaped fields and actual patient motion data are needed for clinical use to provide the data that are sufficient for verification.

## V. CONCLUSIONS

We proposed simple QA of the tracking accuracy for Vero4DRT system using a light field and a web camera. As the web camera has a low cost, this QA method provides a convenient way to verify the tracking accuracy. Our method can assess the tracking accuracy using a light field without irradiation and clearly visualize distributions like film dosimetry.

## COPYRIGHT

This work is licensed under a Creative Commons Attribution 3.0 Unported License.
